# Notes and News

**Published:** 1902-09-27

**Authors:** 


### Notes and News.

Cholera continues in Egypt. At Cairo and Alexandria the proportion of fatal cases is very heavy; out of a total of 30,931 cases reported from July 15th to date 25,734 have died. The Riviera rather than Egypt will probably be the favourite winter resort of the well-to-do this year.

Carlsbad has had upwards of 50,000 patients this season. There are some 200 doctors in Carlsbad, and their distribution of the patients amongst the various springs so as to minimise overcrowding is most ingenious. Apart from the waters the hills and forests and the life and diet would do good to most town residents. Unfortunately, for some unexplained reason, the organisation of the baths in some departments of the Curhaus leaves much to be desired.

Mr. Harry Johnson has been selected out of 151 candidates for the post of secretary to the Portsmouth Hospital. Mr. Johnson has been chief clerk to the secretary of St. Mary's Hospital, Paddington, and was previously in a solicitor's office. The Royal Portsmouth Hospital has become a by-word amongst provincial institutions, so that the task before Mr. Johnson is not a light one, if its management and finances are to be brought up to modern standards. Mr. Johnson is, however, young enough to welcome difficulties of this kind, and we hope for his own sake and for the sake of the suffering poor of Portsmouth that his appointment may prove in every way a success.

The correspondence in a contemporary deals with the abuse of hospitals by the well-to-do. As the metropolitan and other hospitals have increased in efficiency, their popularity has risen with all classes. It is generally recognised that when ill the best place to enter is a hospital. For these reasons hospital managers will soon find themselves on the horns of a dilemma. Either they must face unpopularity by taking effectual steps to exclude from the free beds everybody who can afford to pay, or they must organise separate pay wings for the reception of paying patients. The latter course is the proper one, and those hospital managers who are prompt to recognise the fact will have good cause to congratulate themselves in the near future.

A project has been started in France to buy the house in which Pasteur was born and present it to the town of Dôle.

Thirteen French officials succumbed to yellow fever during the epidemic at Grand Bassam, which has now completely subsided.

Lord Salisbury, who has been seriously ill at Lucerne, is making satisfactory progress towards recovery, but will, it is expected, be unable to travel for another fortnight.

A movement is spreading in favour of legislation entrusting to county councils the duty of providing additional idiot asylums and homes for defective epileptics.

Sir Conan Doyle has given £1,000 to the University of Edinburgh to institute a bursary in connection with the Faculty of Medicine, available for students in South Africa.

The Cremation Act, 1902, has received the royal assent. It confers powers on burial authorities to erect and maintain crematoria subject to the plans of the latter being first approved by the Local Government Board.

The new departure at the Birmingham General Dispensary, where the resident medical officers have been strengthened by the addition of eleven paid visiting consultants will be watched with interest. We welcome this experiment, being convinced that in due course eleemosynary medical service will be everywhere abolished. Free medical attendance is unjust to the profession, and adds materially to the cost of hospital maintenance. Hence its speedy abolition is called for in the interests of all parties.

The new building and restoration fund of the Northampton General Infirmary now amounts to approximately £15,000. An urgent appeal is being made to the town and county for a further £15,000, and we have little doubt that the response will be hearty, seeing that the object is undoubtedly one which is calculated to add immeasurably to the comfort and health of the inhabitants. Amongst the principal donors are Lord Spencer, the Marquis of Northampton, Lady Wantage, and the Mayor of Northampton, Mr. F. G. Adnitt, J.P., who has taken the keenest interest in the movement from the outset.

We offer our sympathy to the proprietors of the *Lancet* in the annoyance they have experienced owing to the action of a drug company in using the name of the *Lancet* for a patent medicine. The letters addressed to our contemporary's representative in New Zealand by the drug company in question are evasive and unsatisfactory. Despite the difficulties which they must encounter, having regard to the important issues at stake, we hope the proprietors of the *Lancet* will put the law in action and proceed in the colony of New Zealand against Messrs. Kempthorne, Prosser and Co. The cost and trouble may be great, but the results on public grounds cannot fail to be satisfactory.

